# Impact of Coffee Consumption on Subjective Perception and Inflammatory Markers in Patients with Inflammatory Bowel Diseases

**DOI:** 10.3390/biomedicines12081733

**Published:** 2024-08-02

**Authors:** Lidia Neamți, Simona R. Gheorghe, Amalia Ventuneac, Tudor Drugan, Cristina Drugan, Ciprian N. Silaghi, Lidia Ciobanu, Alexandra M. Crăciun

**Affiliations:** 1 Department of Medical Biochemistry, “Iuliu Hațieganu” University of Medicine and Pharmacy, 400012 Cluj-Napoca, Romania; lidia.neamti@umfcluj.ro (L.N.); gheorghe.simona@umfcluj.ro (S.R.G.); cdrugan@umfcluj.ro (C.D.); silaghi.ciprian@umfcluj.ro (C.N.S.); acraciun@umfcluj.ro (A.M.C.); 2 Department of Internal Medicine, 1 Medical Clinic, “Iuliu Hatieganu” University of Medicine and Pharmacy, 400006 Cluj-Napoca, Romania; 3 Department of Medical Informatics and Biostatistics, “Iuliu Hațieganu” University of Medicine and Pharmacy, 400012 Cluj-Napoca, Romania; tdrugan@umfcluj.ro; 4 Department of Internal Medicine and Gastroenterology, “Prof. Dr. Octavian Fodor”, Regional Institute of Gastroenterology and Hepatology, “Iuliu Hatieganu” University of Medicine and Pharmacy, 400162 Cluj-Napoca, Romania; ciobanulidia@yahoo.com

**Keywords:** inflammatory bowel diseases, Crohn’s disease, ulcerative colitis, coffee, inflammatory markers

## Abstract

Inflammatory bowel diseases (IBD), including Crohn’s disease (CD) and ulcerative colitis (UC), are chronic conditions marked by persistent inflammation, impacting patients’ quality of life. This study assessed differences in coffee consumption between CD and UC patients and its potential effects on the subjective perception and objective changes in inflammation markers in these two categories of patients. Using questionnaires, coffee consumption patterns, and perceived symptom effects were evaluated. Biological samples were collected to measure the following inflammatory markers: leukocytes, C-reactive protein (CRP), erythrocyte sedimentation rate (ESR), and fecal calprotectin (FC). Among 148 patients, 60% reported regular coffee consumption, with no significant difference between CD and UC patients. While 45.93% perceived no impact on symptoms, 48% of those reporting exacerbation continued their regular coffee consumption. FC values were significantly lower in coffee consumers than in non-consumers (*p* < 0.05), particularly in those consuming natural coffee (*p* < 0.001), and the case was observed for UC patients (*p* < 0.05). No significant differences were observed in other inflammatory markers, regardless of coffee type, frequency, or milk addition. This study highlights the commonality of coffee consumption among IBD patients and the association of lower FC levels with coffee consumption, especially in UC patients, suggesting that coffee may influence intestinal inflammatory responses.

## 1. Introduction

Crohn’s disease (CD) and ulcerative colitis (UC) are inflammatory bowel diseases (IBD) with increasing incidence but an incompletely elucidated pathogenesis. In addition to genetic factors and aberrant immune responses, diet is believed to play a vital role in the occurrence of these diseases by inducing specific changes in the intestinal microbiome [1,2].

Research into the link between nutrition and IBD has increased in recent years [2]. Previous research has noted that certain dietary patterns are associated with the onset [3] but also the exacerbation of IBD [4] by inducing and maintaining a pro-inflammatory state in the intestine [5]. Various studies have investigated and identified connections between the pro- or anti-inflammatory effects of various dietary components and markers of inflammation, such as fecal calprotectin (FC) or C-reactive protein (CRP) and the erythrocyte sedimentation rate (ESR) [6]. FC is a marker of intestinal inflammation that significantly correlates with endoscopic disease activity in IBD, providing an essential tool for assessing endoscopic activity and remission, particularly in UC patients [7]. CRP and ESR are among the most used systemic inflammatory markers. Even if they show a modest correlation with endoscopic activity, they can be associated with therapeutic response, especially CRP [8].

Coffee is one of the main beverages consumed worldwide, which is why it has gained increased interest in analyzing its effects in various conditions. Coffee is a mixture of over a thousand different chemicals. While the effects of many coffee compounds on the body are not fully understood due to their relatively low concentrations, three are recognized for their significant bioactive effects: caffeine, diterpenic alcohols (cafestol and kahweol), and chlorogenic acid, along with other polyphenols [9]. Among the properties attributed to these compounds are antioxidant and anti-inflammatory effects [10]. Thus, the beneficial effects of regular coffee consumption have been described for various chronic diseases involving different degrees of inflammation, such as liver disease [11], type 2 diabetes [12], neurodegenerative diseases [13], constipation [14], or cancer [15]. Moreover, regular coffee consumption has been linked to a decrease in general mortality, but especially when related to diabetes or cardiovascular, respiratory, and infectious conditions [16]. Regarding the effect of coffee on the intestine, it has been observed that coffee consumption can increase the production of short-chain fatty acids at the intestinal level, thus having a probiotic effect [17]. Other observed effects include the stimulation of colonic motor activity [18] or increased rectal tone [19]. Some studies have also observed a protective effect against the development of UC [20,21]. These results were not dependent on caffeine content, suggesting that caffeine may not be responsible for the beneficial effects [22]. However, prolonged coffee consumption can also have negative effects, such as increased blood pressure values, predominantly in the elderly [23], or the alteration of sperm quality, possibly leading to infertility [24].

Due to the prokinetic effects on the digestive tract [25], coffee consumption is often not recommended for patients with IBD, especially during the active state of the disease [26]. However, despite the laxative effect of coffee, the anti-inflammatory compounds it contains may still have a positive impact in reducing the inflammation associated with these conditions. Although several studies have evaluated coffee consumption among patients with IBD [21,27,28,29,30], to date, no study has examined the relationship between this habit and various markers of inflammation used in clinical practice. In addition, the available studies on this topic are highly heterogeneous, which underlines the need for more extensive research on the effects of coffee consumption in order to evaluate its impact on quality of life and to provide evidence-based recommendations.

Considering the large number of coffee consumers among IBD patients [27], it is essential to understand the effects and consequences of this practice on these conditions. Therefore, this study aims to investigate the differences in coffee consumption between patients with CD and UC and to determine its potential influences on the subjective perception and objective changes of inflammatory markers in these two patient categories.

## 2. Materials and Methods

The study was conducted within the Regional Institute of Gastroenterology and Hepatology Cluj-Napoca, Romania, for a period of 6 years between 2017 and 2022. The included patients had clinically, endoscopically, and histologically confirmed diagnoses of CD or UC. The patients were selected randomly. All the patients who met the inclusion criteria and consented to undergo a colonoscopy were asked to participate in the study. Patients gave their informed consent to participate. The endoscopist only performed an intestinal biopsy in cases considered necessary.

Clinical and demographic data collected included the type of disease, age at inclusion in the study, age at diagnosis, gender, and smoking status. Patients with other conditions that could have influenced the inflammatory status, such as neoplasms, infections, and autoimmune diseases, or pregnant women were excluded. Depending on clinical, endoscopic, and histological activity status, based on internationally validated scores widely used in current practice, patients were assigned to one of the following subgroups: active disease and disease in remission [31]. Thus, to classify patients with CD, the Crohn’s disease activity index (CDAI) was used to evaluate clinical activity, a simple endoscopic score (SES-CD) was used to evaluate endoscopic activity, and the Nani and Cortina score was applied for histological activity. Patients with a CDAI score of <150 points, an SES-CD score of <2 points, and a histological score of <2 points were classified in the remission subgroup. To classify patients with UC, the partial Mayo score was used to evaluate clinical activity, the Mayo endoscopic score was applied for the assessment of endoscopic activity, and the Nancy score was utilized for the evaluation of histological activity. A partial Mayo score of <2 points, an endoscopic Mayo score = 0 points, and a Nancy score < 1 point were considered suggestive of the disease in remission. The other patients were classified as having active disease.

Coffee consumption was assessed by completing a questionnaire previously validated on a similar population within the same hospital. Patients were asked to complete the questionnaire by entering data from the last month before collection. The questionnaire included questions about the type of coffee consumed, the frequency or the amount of consumption, the possible addition of/amount of milk or other similar products, and the eventual addition, amount and type of sweeteners. We proposed categorizing coffee types as follows: natural coffee, which refers to coffee that has been minimally processed and is closer to its natural state; soluble coffee, which is brewed coffee that has been freeze-dried or spray-dried into granules or powder for quick preparation; decaf coffee, which has most of its caffeine removed; cappuccino, a blend of equal parts espresso, steamed milk, and milk foam and other types. Measures commonly used in the kitchen were used to estimate quantities (e.g., cup, spoon). In addition, the patient’s perception of coffee consumption (“Does coffee have a beneficial effect on health?”), and the impact of coffee consumption on symptoms (does it improve/worsen/does not influence the symptoms or the patient/ does not know) were also evaluated.

Systemic inflammation was evaluated in serum, in the morning, in fasting condition, by leukocyte count (cells/µL), ESR (mm/h), and CRP (mg/dL). Intestinal inflammation was evaluated by measuring FC (µg/g). All inflammatory markers analyzed were quantified in the hospital laboratory. The intra-assay CV for each parameter was <10%. Thus, the number of leukocytes was counted by electrical impedance and light scattering using an automated analyzer Sysmex XN 1000 (Sysmex, Kobe, Japan); capillary photometry was used for ESR measurement on Alifax TEST1 (Alifax, Polverara, Italy), turbidimetric immunoassay was used for CRP determination on Cobas c503 (Roche Diagnostics, Rotkreuz, Switzerland) and an ELISA kit, calprotectin (Orgentec Diagnostika, Mainz, Germany) was used for FC determination. The assay sensitivity was 17.5 µg/g with an intra-assay precision of <6%.

Statistical analysis was performed using the SPSS program (version 25; IBM Corp., Armonk, NY, USA). Clinical and demographic data were presented as the mean ± standard deviation (SD), median, and interquartile range or as percentages (%). Testing for the normality of data distribution was performed by the Kolmogorov–Smirnov test, and differences between groups were evaluated by the Mann–Whitney U test in the case of non-normally distributed quantitative variables and by the Chi-square test or Fisher’s exact test in the case of qualitative variables, respectively. Due to the low number of participants and the fact that quantitative variables were not normally distributed, we did not perform a power analysis. Values of *p* < 0.05 were considered statistically significant.

## 3. Results

**Figure 1:**
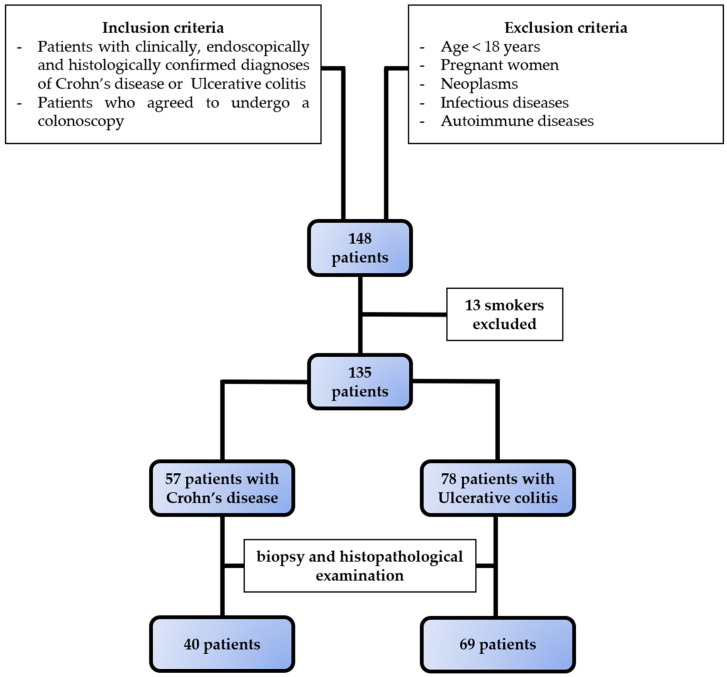
Selection of study population. Based on the inclusion and exclusion criteria, we selected 57 patients with Crohn’s disease and 78 patients with ulcerative colitis.

The selection of subjects is presented in Figure 1, and the clinical and demographic characteristics of the patients are shown in Table 1. The onset of CD was at a younger age than that of UC. There were no differences in patient gender between the two diseases.

Regarding disease activity, 57.04% of patients were in a state of clinical activity, 85.37% had endoscopic activity, and 92.66% had histological activity. The disease-specific activity classification is shown in Table 1. Statistically significant clinical activity differences were observed between the two diseases, without differences regarding endoscopic or histological activity.

**Figure 2:**
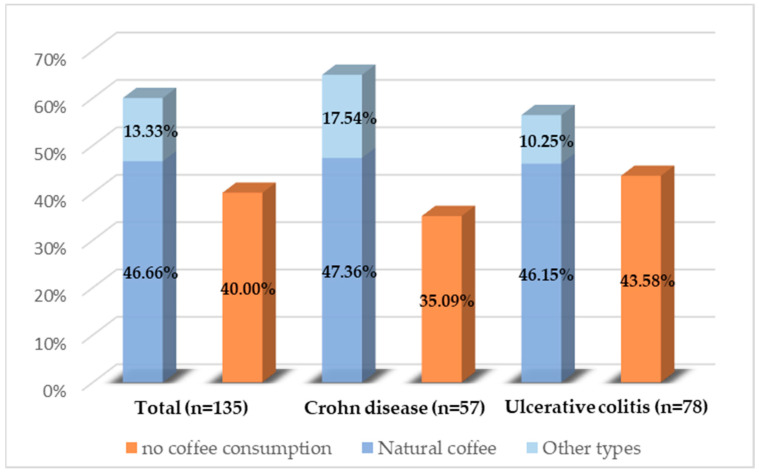
Coffee consumption distribution among the study population.

Of the 135 patients analyzed, all completed the questionnaire in full. Of these, 60% reported regular coffee consumption. No statistically significant differences were identified regarding the proportion of patients consuming coffee between the two diseases (*p* = 0.31) or between the sexes (*p* = 0.44). Figure 2 illustrates the types of coffee consumed. Natural coffee was the predominant choice, while other types, such as soluble coffee, decaffeinated coffee, or cappuccino, were less frequently chosen. There were no significant differences in the type of coffee consumed between the sexes.

In terms of coffee consumption frequencies, 28.14% of all participants reported daily consumption. Additionally, 40.74% of respondents indicated adding milk to their coffee. Patients diagnosed with CD reported significantly higher rates of milk-added coffee consumption compared to those with UC (*p* < 0.001). Among all respondents, 15.15% added one spoon of milk, 63.64% added between two and four spoons, and 21.21% added more than four spoons. Regarding the use of sweeteners in coffee, 56.79% reported frequent addition, with no significant difference observed between CD and UC patients (*p* = 0.07). Within this cohort, 67.39% used white sugar, 17.39% used brown sugar, 8.70% used other types of sweeteners (e.g., stevia, saccharin, honey), and 6.52% used mixtures of sweeteners.

All patients (consumers and non-consumers of coffee) were asked about their general opinion regarding the effect of coffee on symptomatology. In total, 45.93% of patients with IBD declared that coffee consumption did not influence their symptoms; this percentage was slightly higher in CD, compared to UC (50.87% vs. 42.30%), without statistically significant differences when applying the Chi-square test (*p* = 0.40). The percentage of patients who reported the worsening of symptoms due to coffee consumption was slightly lower in CD, compared to UC (17.54% vs. 19.23%), while the proportion of patients who reported an improvement in symptoms was relatively equal between the two diseases (5.26% in CD and 5.12% in UC, respectively). However, when applying the Chi-square test, no statistically significant differences between the two diseases were found with respect to symptom aggravation (*p* = 0.80) or improvement (*p* = 0.97). A significant number of participants (30.37%) declared that they did not know if coffee consumption influenced their symptoms. Most of them belonged to the group who never consumed coffee. There were no significant differences between the sexes in the perception of coffee’s effects on symptomatology.

Among the reasons why some patients avoided drinking coffee were the doctor’s recommendation to avoid coffee, not feeling the need to drink coffee, or the worsening of symptoms in cases of consumption. The main symptoms associated with coffee consumption were increased stool numbers, sometimes associated with decreased consistency or worsening abdominal pain. However, 48% of the patients who reported worsening symptoms associated with coffee consumption did not give up this habit. It should be noted that five patients declared that they avoided coffee consumption only during periods of clinical activity related to the disease.

The patients were asked about their perception of the relationship between coffee consumption and its harmful effects on health. Of the total participants, 51.11% claimed that coffee consumption has no harmful effects, 17.78% claimed that coffee consumption has a negative effect on health, regardless of the amount consumed, 3.70% mentioned that this negative effect occurs only when consuming increased amounts, and 27.41% declared that they did not know whether coffee consumption influences the state of health. Among those who answered yes or did not know, 42.42% were regular coffee consumers.

Next, the connection between coffee consumption and the state of disease activity was evaluated using specific scores. No statistically significant differences were found between coffee-consuming and non-consuming patients for clinical activity (*p* = 0.67), endoscopic activity (*p* = 0.22), or histological activity (*p* = 0.61).

The influence of coffee was objectively evaluated by analyzing inflammation markers according to the type of disease and the coffee consumer/non-consumer status. The results are shown in Table 2 using the mean ± standard deviation for normally distributed variables and the median and interquartile range with the 25% and 75% percentiles for variables with a non-normal distribution. Within the whole group of patients, the FC values were significantly lower in patients who consumed coffee compared to those who did not (*p* = 0.04). However, this statistical significance was not maintained for each type of disease. No statistical significance was obtained when comparing the values of inflammatory markers according to the type of coffee, the frequency of its consumption, or the addition of milk. Regarding the addition of sweeteners, significantly lower ESR-2h values were observed in patients who consumed coffee without sweeteners compared to those who added sweeteners (*p* = 0.03).

Finally, in order to investigate whether the effect of coffee is attributable to caffeine or other compounds it contains, we analyzed whether any statistical differences in inflammatory markers can be found between patients who consumed different types of coffee. The results did not reveal statistically significant differences between instant coffee consumers and those who did not drink coffee or those who consumed other types of coffee. However, there was a significant difference between FC values in patients who exclusively consumed natural coffee and those who did not consume coffee at all, both in the overall patient sample (*p* < 0.001) and in patients with UC (*p* = 0.03), but not in CD patients (*p* = 0.15). Table 3 presents the values of inflammatory markers according to disease type and whether patients were natural coffee consumers or non-consumers. For normally distributed variables, the values are reported as the mean ± standard deviation, while for non-normally distributed variables, they are reported as median and interquartile range, including the 25th and 75th percentiles.

## 4. Discussion

This study aimed to assess coffee consumption patterns among individuals with IBD by examining their subjective experiences regarding coffee’s impact on their symptoms. Additionally, it aimed to objectively analyze inflammatory markers in this population in order to elucidate their potential associations with coffee consumption.

Over the years, numerous studies have investigated the influence of various environmental factors on the progression of IBD [32]. Recently, more emphasis has been placed on the role of nutrition in the pathogenesis of IBD and its potential impact on symptomatology [33]. Despite coffee being one of the most commonly consumed beverages globally and containing compounds known for its anti-inflammatory properties, its effects on IBD remain largely unexplored. To the best of our knowledge, this study represents the first attempt to investigate the impact of coffee consumption on inflammatory markers in individuals with IBD.

The decision to exclude smokers from the statistical analysis was motivated by smoking’s well-known pro-inflammatory impact [34] and its paradoxical protective effect observed in patients with UC [35]. This exclusion was necessary to mitigate potential confounding effects on the study outcomes.

In our study, the number of patients exhibiting endoscopic or histological activity surpassed those reporting clinical symptoms. This observation aligns with findings from previous research, which demonstrates that intestinal lesions may not always become clinically manifest [36,37].

Coffee consumption has globally experienced a steady rise in recent decades. Europe currently leads the world in coffee consumption, averaging 5 kg per person per year [38]. Our study found that 60% of patients regularly consume coffee, with no significant variance observed between those diagnosed with CD and UC. Most patients reported daily coffee consumption, which is a pattern consistent with data from the United States, where approximately 65% of the population drinks coffee daily [39]. Preference for caffeinated coffee was predominant among participants, with only a tiny fraction opting for decaffeinated varieties, mirroring findings from a previous study on IBD patients’ coffee-drinking habits [27]. This choice likely stems from individual taste preferences and the widespread belief in caffeine’s potential to enhance cognitive function and mood [40]. Additionally, a notable observation was that patients with CD tended to add milk to their coffee more frequently than patients with UC. While this aspect lies beyond the scope of our study, it may be speculated that dietary preferences among IBD patients, who often avoid foods that exacerbate symptoms, could contribute to this trend [30]. Consequently, dairy consumption might have a more pronounced negative impact on UC patients compared to CD patients. However, it is crucial to acknowledge that in our study, a higher proportion of UC patients were experiencing clinical disease activity, which could further influence their dietary choices.

In contrast to the study by Barthel et al. [27], our investigation did not reveal statistically significant disparities between the two diseases in the subjective perception of coffee’s effect on symptomatology. Nevertheless, a noteworthy finding was that most patients, across both conditions, who attributed coffee to an effect on symptomatology reported a negative impact. Intriguingly, this negative perception did not translate into complete abstention from coffee consumption. Such findings highlight the inconsistencies found in the existing literature regarding the relationship between coffee intake and IBD. Some studies suggest a protective role of coffee due to its anti-inflammatory [41] or anti-neoplastic properties [15]. Moreover, coffee consumption has not been linked to an increased incidence of IBD [28]; instead, it has been deemed protective [21]. Conversely, other research indicates potential pro-inflammatory effects of coffee on the healthy intestine, such as the activation of the NF-kB transcription factor [42], as well as stimulatory effects on intestinal motility, that could influence symptomatology in patients with functional bowel disorders [25]. Moreover, studies indicate that individuals with CD and UC tend to consume less coffee compared to healthy counterparts [29]. Despite negative perceptions or personal beliefs regarding coffee’s harmful effects, the reason why many patients persist in its consumption remains elusive. Speculatively, perceived effects on disease progression may not be significant enough to warrant complete avoidance. Alternatively, the negative impact of coffee could be downplayed or even ignored, indicating characteristics of addiction. Additionally, some patients may be swayed by emerging evidence supporting the health benefits of coffee [11,12,13,14,15,16]. However, it is noteworthy that certain patients reported refraining from coffee consumption only during periods of clinical activity, suggesting selective avoidance of certain foods during specific disease stages, which is a finding supported by other studies [30].

Regarding the impact of coffee consumption on inflammatory markers, numerous studies have explored its influence on serum levels of CRP and various cytokines, including interleukins (ILs), such as IL-6, IL-8, and tumor necrosis factor (TNF)-alpha [43]. However, to date, no investigations have examined this relationship, specifically in patients with IBD. Consistent with previous research on serum CRP changes associated with coffee intake, our study did not reveal significant differences between coffee-consuming and non-consuming patients [44,45]. Similarly, no significant alterations were observed in other markers of systemic inflammation, such as leukocyte count or ESR-2h. Nonetheless, FC levels, a marker of intestinal inflammation, were lower among regular coffee consumers. This reduction could be attributed to the mitigating effects of coffee on acute colitis at the level of the intestinal epithelial cells, as previously demonstrated following oral caffeine administration [46]. The absence of consistent effects across different forms of IBD subgroups could be due to the relatively small sample size within each subgroup. Furthermore, our findings suggest that the association between coffee consumption and reduced FC values was significant for natural coffee drinkers rather than consumers of other coffee types. This result implies that the observed effect may be linked, at least partially, to specific compounds present in natural coffee, distinct from caffeine alone, for example, flavonoids, which are known for their fight against reactive oxygen species [47]. While no significant differences were detected between non-consumers and those preferring other types of coffee, our results underscore the importance of examining the precise composition of various coffee types and their effects on inflammatory markers in diverse conditions. Interestingly, the association between natural coffee consumption and FC levels persisted within the UC patient subgroup, hinting at the potential benefits of coffee intake for this particular condition. However, the absence of a significant similar association among CD patients underscores the necessity for further investigation to better understand coffee’s impact on this subgroup.

Moreover, the addition of milk to coffee did not yield notable changes in inflammatory markers, likely due to the limited number of patients who reported this habit and possibly driven by concerns about dairy exacerbating symptoms, which is an assumption supported by other studies [30]. Nonetheless, the type of milk used goes beyond the scope of our study.

On another note, the addition of sweeteners influenced ESR-2h values, with lower levels observed in non-sweetener users. Although sugar and artificial sweeteners are generally regarded as safe, animal studies suggest that they may contribute to gastrointestinal inflammation [48]. Surveys of IBD patients indicate that between 10% and 36% perceive high-sugar foods as exacerbating their symptoms and triggering flare-ups [49].

The present study has several limitations. Firstly, the small sample size is a significant constraint, which can be attributed to the low prevalence (1.5 per 100,000 for CD and 2.4 per 100,000 for UC, respectively) and incidence rates (1.7 per 100,000 people/year for CD and 2.5 per 100,000 people/year for UC) of IBD in Romania, as well as the limited availability of patients in a single regional center [50]. Consequently, our findings may only partially represent the broader population of individuals with IBD. In addition, to ensure a robust participation rate and gather a substantial number of responses, this study focused on patients’ perspectives regarding coffee consumption and its perceived effects on symptomatology. Consequently, other relevant factors, such as alcohol consumption, tea intake, or the consumption of other caffeinated beverages, were not explored. A more comprehensive approach, encompassing a broader range of dietary habits, could have provided richer insights into patients’ overall eating behaviors. Additionally, it is essential to acknowledge that a questionnaire-based study has inherent limitations and cannot substitute for a clinical trial. The lack of detailed data regarding coffee processing, brewing methods, precise consumption quantities for coffee, and other additives prevented a thorough analysis of coffee’s impact. More rigorous methodologies and detailed data collection would have been necessary to address these aspects, which were not feasible within the scope of this study.

The findings of our study underline the need for further research into this realm to validate and expand upon our observations regarding the influence of coffee consumption on individuals with IBD. Clinical trials play a pivotal role in furnishing robust evidence and shaping evidence-based recommendations for patient care. Moreover, it is crucial to acknowledge that some individuals may refrain from coffee consumption, either because of their own convictions or under the guidance of their healthcare provider, thus highlighting the necessity for an individualized approach to managing IBD. Continued investigation may elucidate who can benefit from coffee consumption and under what circumstances, risks, or contraindications this practice is associated with. By delving deeper into these aspects, we can refine our understanding of how coffee interacts with IBD and tailor therapeutic approaches to optimize patient outcomes.

## 5. Conclusions

In conclusion, while most patients reported regular coffee intake, a noteworthy proportion identified coffee as a potential influencer of intestinal symptoms. Intriguingly, despite this negative perception, many continued to consume coffee. Although systemic inflammatory markers remained largely unaffected by coffee consumption, a correlation emerged between regular coffee intake and reduced levels of FC, especially for patients who consumed natural coffee and had UC, suggesting the potential mitigating effect of coffee on intestinal inflammation. This discrepancy underlines the need for further investigation into the specific components and mechanisms regarding coffee’s impact on IBD pathophysiology. Such endeavors are crucial for formulating evidence-based recommendations and guiding medical management strategies tailored to the needs of patients with IBD.

## Figures and Tables

**Table 1:** Clinical and demographic characteristics of the included subjects.

	CD *n* = 57	UC *n* = 78	*p* Value
**Male, n (%)**	28 (49.12%)	39 (50.00%)	0.92
**Age at inclusion (years)**	37.81 ± 13.50	44.05 ± 16.21	0.01
**Age at diagnosis (years)**	31.97 ± 9.92	40.38 ± 16.04	0.03
**Disease activity**			
**Clinical, n (%)**	19 (33.33%)	58 (74.36%)	<0.001
**Endoscopic, n (%)**	39 (78.00%)	66 (90.41%)	0.05
**Histological, n (%)**	36 (90.00%)	65 (94.20%)	0.46

Depending on the distribution, the data are presented as the mean ± SD or number (percentage), as appropriate. Abbreviations: CD, Crohn’s disease; UC, ulcerative colitis; n, number of subjects.

**Table 2:** Inflammatory markers according to disease type and coffee consumer/non-consumer status.

	Total *n* = 135		CD *n* = 57		UC *n* = 78	
Coffee Consumption	No	Yes	No	Yes	No	Yes
**Leukocyte** cells/µL	7933.21 ± 2496.55	8057.28 ± 2779.64	7997.37 ± 2408.56	8501.62 ± 3194.21	7897.35 ± 2579.40	7683.64 ± 2349.46
**ESR2h** mm/h	44 (22–81)	40 (20–78.50)	52 (36–99)	56 (20–81)	40 (18–76)	40 (21–74)
**CRP** mg/dL	0.78 (0.4–3.52)	0.63 (0.39–3.12)	0.96 (0.41–6.50)	0.86 (0.41–4.84)	0.63 (0.39–3.08)	0.53 (0.39–2.13)
**FC** µg/g	1143 * (300–2100)	611.75 * (163.75–1754.25)	798 (175.40–2100)	548.50 (185–1247.75)	1400 (400–2100)	754.50 (126–1957)

Depending on the distribution, the data are presented as the mean ± standard deviation or median (interquartile range–25th and 75th percentile) as appropriate. Abbreviations: CD, Crohn’s disease; UC, ulcerative colitis; n, number of subjects; ESR2h, two-hour erythrocyte sedimentation rate; CRP, C-reactive protein; and FC, fecal calprotectin. Statistically significant differences are marked with *.

**Table 3:** Inflammatory markers according to disease type and natural coffee consumer versus non-consumer status.

	Total *n* = 116		CD *n* = 47		UC *n* = 70	
	No Coffee	Natural Coffee	No Coffee	Natural Coffee	No Coffee	Natural Coffee
**Leukocyte** cells/µL	7933.21 ± 2496.55	7960.95 ± 2510.66	7997.37 ± 2408.56	8354.44 ± 2908.87	7897.35 ± 2579.40	7665.83 ± 2161.32
**ESR2h** mm/h	44 (22–81)	40 (20–78.50)	52 (36–99)	56 (20–81)	40 (18–76)	40 (22–76)
**CRP** mg/dL	0.78 (0.4–3.52)	0.53 (0.4–2.14)	0.96 (0.41–6.50)	0.63 (0.41–3.26)	0.63 (0.39–3.08)	0.50 (0.39–1.48)
**FC** µg/g	1143 * (300–2100)	790.99 * (144–1441.50)	798 (175.40–2100)	463.50 (175–1214)	1400 * (400–2100)	623.50 * (99–1800)

Depending on the distribution, the data are presented as the mean ± standard deviation or median (interquartile range—25th and 75th percentile) as appropriate. Abbreviations: CD, Crohn’s disease; UC, ulcerative colitis; n, number of subjects; ESR2h, two-hour erythrocyte sedimentation rate; CRP, C-reactive protein; and FC, fecal calprotectin. Statistically significant differences are marked with *.

## Data Availability

Data presented in this study are available upon reasonable request from the corresponding author. The data are not publicly available due to privacy regulations.
